# Combining Prognostic Nutritional Index and Brain Natriuretic Peptide as a Predicting Tool for Heart Transplantation

**DOI:** 10.3390/jcdd9020040

**Published:** 2022-01-24

**Authors:** Ziwen Cai, Jingrong Tu, Li Xu, Yao Lin, Bowen Deng, Fei Li, Si Chen, Nianguo Dong

**Affiliations:** 1 Department of Cardiovascular Surgery, Union Hospital, Tongji Medical College, Huazhong University of Science and Technology, Wuhan 430022, China; caiziwen@hust.edu.cn (Z.C.); tjrforwork@126.com (J.T.); 2021507078@hust.edu.cn (L.X.); lifei_union@hust.edu.cn (F.L.); 2 Department of Gastrointestinal Surgery, Union Hospital, Tongji Medical College, Huazhong University of Science and Technology, Wuhan 430022, China; linyaomt@163.com; 3 The First Clinical College, Tongji Medical College, Huazhong University of Science and Technology, Wuhan 430022, China; u201810271@hust.edu.cn

**Keywords:** heart transplantation, prognostic nutritional index, brain natriuretic peptide, propensity score matching, China

## Abstract

Our study aimed to evaluate the potential of prognostic nutritional index (PNI) and Brain natriuretic peptide (BNP) in predicting the prognosis of heart transplantation (HTx). We retrospectively investigated 489 patients undergoing HTx between 2015 and 2020 in our center. The relationship between preoperative index and prognosis was analyzed respectively, the optimal cut-off values for preoperative PNI and BNP level were evaluated with receiver operating curve analysis. Uni-variate analysis and multivariate analysis were used to compare baseline data (sex, age, diagnosis, etc.) of groups divided by the level of PNI and BNP. Propensity score matching (PSM) was applied to eliminate bias. We calculated the C-index from the prediction efficiency of PNI and BNP. During the period, 489 recipients undergoing HTx in our center were included according to the inclusion criteria; 383 (78.3%) males and 106 (21.7%) females were included in this study, with a median age of 47.57 years old. The ROC curve showed that the optimal cut-off values of each indicator were verified as 49.345 for PNI, and 4397.500 for BNP. The multivariate analyses indicated that PNI (*p* = 0.047), BNP (*p* = 0.024), age (*p* = 0.0023), and waiting time (*p* = 0.012) were risk factors for all-cause death after HTx. Propensity score matching generated 116 pairs based on PNI level and 126 pairs based on BNP level, and the results showed that OS (overall survival) was significantly correlated with PNI (*n* = 232, *p* = 0.0113) and BNP (*n* = 252, *p* = 0.0146). Our study implied that higher PNI and lower BNP level had direct correlation with better survival after HTx. Combining PNI and BNP together would be a potential clinical preoperative instrument to predict the survival of patients after HTx, especially in short-term survival.

## 1. Introduction

Heart transplantation (HTx) remains the “gold standard” procedure for patients with end-stage heart failure refractory to other treatments. The registry of International Society of Heart and Lung Transplantation (ISHLT) demonstrated the median survival in adult recipients between 2002 and 2009 was 12.5 years, and it increased to 14.8 years among 1-year survivors (ISHLT2019). The causes for short-term death includes acute graft failure, infectious complications, acute rejection, and renal failure. Moreover, the number of people on the waiting list for HTx far exceeds the number of people who will actually undergo HTx. Therefore, it is extremely necessary to identify the clinical and demographic characteristics of recipients, and find out the factors that could help to predict the prognosis of HTx.

Heart-failure-related inflammation might bring about heart dysfunction including ventricular remodeling, cellular metabolic disorder, and cardiomyocyte necrosis. It is essential to investigate some valuable blood biomarkers that can be a tool for prognosis assessment after HTx. Brain natriuretic peptide (BNP) was recommended to be the biomarkers for diagnosis and prognosis of HF in the European clinical practice guidelines [1]. The prognostic nutritional index (PNI) reflects nutritional conditions of the human body and previous studies have demonstrated that PNI could be utilized as a useful indicator to predict prognosis in some cardiovascular diseases such as acute heart failure, coronary artery disease, and myocardial infarction [2,3,4]. However, no previous studies have focused on the relationship between PNI and the prognosis of HTx.

Accordingly, the aim of the present study was to investigate the prognostic values of BNP and PNI in HTx.

## 2. Materials and Methods

### 2.1. Study Population

All consecutive recipients of orthotopic heart transplantation (*n* = 581) were incorporated in this analysis at our center between 1 January 2015 and 31 December 2020. Multiple organ transplantation, re-transplantation, pediatric patients, and recipients with data missing were excluded (*n* = 92), and 489 patients were included in this study. Baseline demographic, clinical, and biochemical data for each patient were retrieved from electronic medical records. The Charlson Index based on a selected number of chronic diseases was used for general assessment of comorbidity status. Follow-up information was obtained for all survivors either through outpatient visit or by telephone interviews with the patients/their relatives, and was complete until 26 May 2021.

### 2.2. Follow-Up Data and Variable Definitions

Regular medical follow-up data were obtained using telephone calls, clinic visits, Internet, and other interaction tools. The all-cause overall survival (OS) rate was defined as the duration from transplantation surgery to the mortality event or the end of follow-up. Mortality data were obtained from China Heart Transplant Registration Network, where all deaths are registered, as required by law.

The demographic data included the sex, age, Body Mass Index (BMI), diagnosis, blood type, heart surgery history, Charlson Comorbidity Index, and waiting time on the list. The recipient/donor indicators included BMI, age, sex, and blood type. The pre-operative treatments included intra-aortic balloon pump (IABP), Cardiac Resynchronization Therapy Defibrillator (CRTD), extracorporeal membrane oxygenation (ECMO), ARB drug, ACEI drug, dopamine, and BB. The pre-operative laboratory indicators included white blood cell count (WBC), red blood cell count (RBC), and levels of blood platelet (PLT), hemoglobin (Hb), glutamic oxaloacetic transaminase (AST), and alanine transaminase (ALT), D-dimer, creatinine (Cr), troponin, brain natriuretic peptide (BNP), triglyceride (TG), and LDL. All pre-operative laboratory examinations were performed within 7 days before surgery. PNI was calculated as 10 × serum albumin (g/dL) + 0·005 × lymphocyte count (per mm^3^).

### 2.3. Statistical Analysis

Categorical variables were presented as count of patients (percentage) and continuous variables as median (interquartile range (IQR)) or mean ± standard deviation (SD), as appropriate. Descriptive comparisons were performed with Pearson’s c2 for categorical variables and Mann–Whitney U rank sum test for continuous variables, as appropriate. Propensity score matching (PSM) was conducted on the basis of 19 clinically relevant variables. The quality of the matching was assessed by absolute standard differences, with a value <5% considered as not significant. Univariate and multivariate Cox proportional hazard modeling was used to estimate the adjusted hazard ratio (HR) and 95% confidence interval (CI) for clinical factors in OS. Survival analysis was generated with the Kaplan–Meier (KM) method, and differences between groups were examined with the log-rank test. For validating the discrimination of PNI and BNP in OS, we calculated the area under the ROC curve (AUC) in the cohort. Furthermore, the Harrell’s C index was generated for discrimination of PNI and BNP using 1000 times bootstrap. Statistical significance was defined by a *p* value of less than 0.05 (two-sided). Analysis was performed using R version 4.0.1 with the packages MatchIt and SPSS 26.0.

## 3. Results

**Figure 1:**
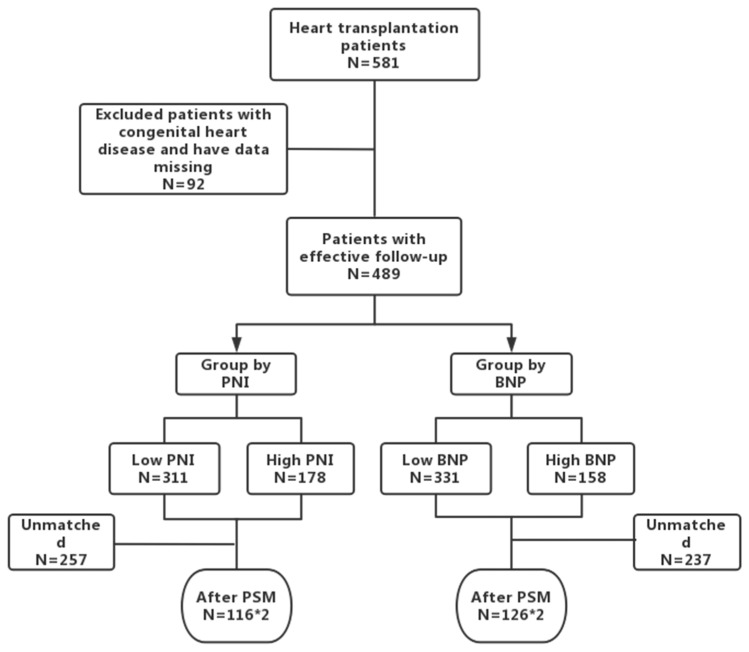
Study cohort. *2 means there were some pairs of patients after PSM.

Our study enrolled 489 patients according to the inclusion criteria (Figure 1 study flow chart); 383 (78.3%) males and 106 (21.7%) females were included in this study, with a median age of 47.57 years old. Among all patients, 95 patients were diagnosed as ischemic cardiomyopathy, 308 patients were diagnosed as non-ischemic cardiomyopathy, 19 were diagnosed as congenital heart disease, and 67 were diagnosed as other heart diseases (valvular cardiomyopathy and arrhythmic cardiomyopathy) (Table 1).

### 3.1. The Optimal Cut-Off Values of PNI and BNP for Estimating Prognosis

**Figure 2:**
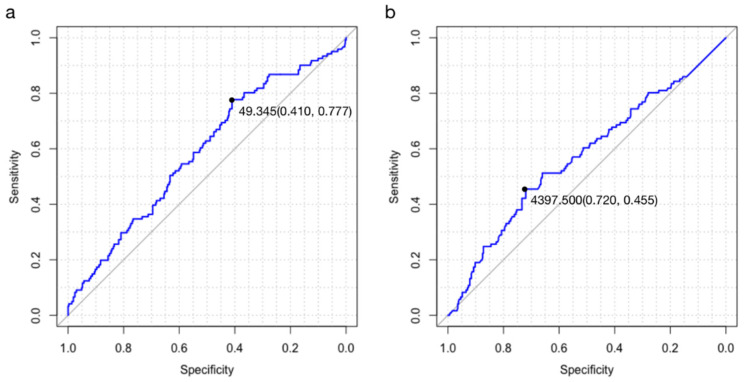
ROC curve for cut-off: (**a**) The ROC of PNI. (**b**) The ROC of BNP.

In our study, we tried to obtain the optimal cut-off values of three potential indicators to predict survival with a ROC analysis method (Figure 2). During the process, the areas under the curve (AUC) of survival were 0.588 (*p* < 0.05, 95% CI 0.333–0.555) for PNI, and 0.572 (*p* < 0.05, 95% CI 0.423–0.776) for BNP. According to Youden index, the optimal cut-off values were verified as 49.345 for PNI, and 4397.500 for BNP. 

### 3.2. Baseline Characteristic of Different Groups

Baseline clinical characteristics were shown and compared between two groups of each blood index separately (Table 1). Higher PNI was significantly related with younger age (*p* = 0.005), larger recipient/donor age ratio (*p* = 0.012), shorter waiting time (*p* < 0.001), more preoperative ACEI use (*p* < 0.001), less preoperative dopamine (*p* = 0.013), more preoperative BB use (*p* = 0.035), lower Cr level (*p* = 0.021), higher D-dimer, Hb, RBC, PLT, WBC, and triglyceride (*p* = 0.029, *p* < 0.001, *p* = 0.013, *p* = 0.006, *p* = 0.01, and *p* < 0.001).

In addition, lower BNP was significantly associated with younger age (*p* = 0.029), higher recipient BMI (*p* = 0.02), lower recipient/donor BMI (*p* < 0.016), less preoperative IABP use (*p* = 0.001), more preoperative ACEI use (*p* = 0.009), less preoperative dopamine use (*p* = 0.001), lower Cr level (*p* = 0.024), and higher triglyceride level (*p* = 0.002). There was no significant difference in other variables between two groups.

### 3.3. Univariate and Multivariate Cox Analysis of OS of Patients with HTx

**Figure 3:**
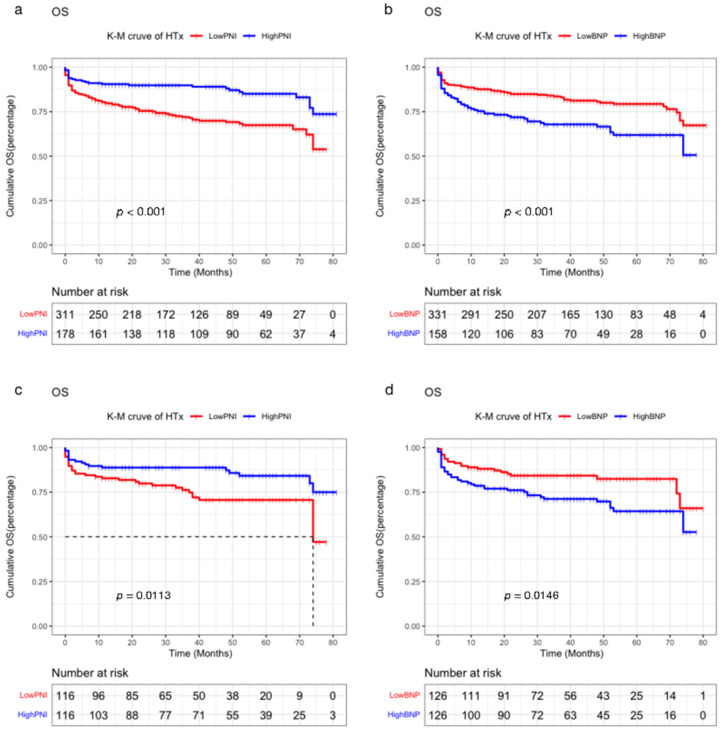
KM curve before and after PSM: (**a**) KM curve of PNI before PSM. (**b**) KM curve of PNI after PSM. (**c**) KM curve of BNP before PSM. (**d**) KM curve of BNP after PSM.

The results presented the relationships between blood biomarkers and OS. The low PNI group had a more significant OS than that of high PNI group (*p* < 0.001, Figure 3a), whereas the high BNP group was observed with worse OS than that of low the BNP group (*p* < 0.001, Figure 3b). Multivariate analysis showed that older age (HR 1.025, 95%CI, 1.009–1.042, *p* = 0.002), low PNI level (HR 0613, 95%CI 0.378–0.993, *p* = 0.047), high BNP level (HR 1.542, 95%CI 1.057–2.248, *p* = 0.024), and long waiting time (HR 1.014, 95%CI, 1.003–1.026, *p* = 0.012) were the independent protective factors for the prognostic of heart transplantation (Table 2).

### 3.4. Survival Analysis of HTx Patients of Different Level of PNI and BNP after PSM

In the PNI cohort, 47.4% (232/489) of patients were successfully matched into pairs, as were 51.5% (252/489) of patients in the BNP cohort. The distribution of propensity score is presented in the Supplementary Materials. The density of propensity score for each arm was shown before and after matching, and those matched represented a balanced and heterogeneous distribution. Unmatched low PNI group and high BNP group had lower scores, whereas unmatched high PNI group and low BNP group had higher scores.

After matching, all baseline characteristics had the differences eliminated, and those variables were equally contributed between both two groups without affecting others for each cohort (Table 3). Kaplan–Meier analysis showed that patients with lower PNI, higher BNP had significantly worse overall survival rates (Figure 3c,d).

### 3.5. Effectiveness Evaluation of BNP and PNI Level in Predicting the OS of HTx

**Figure 4:**
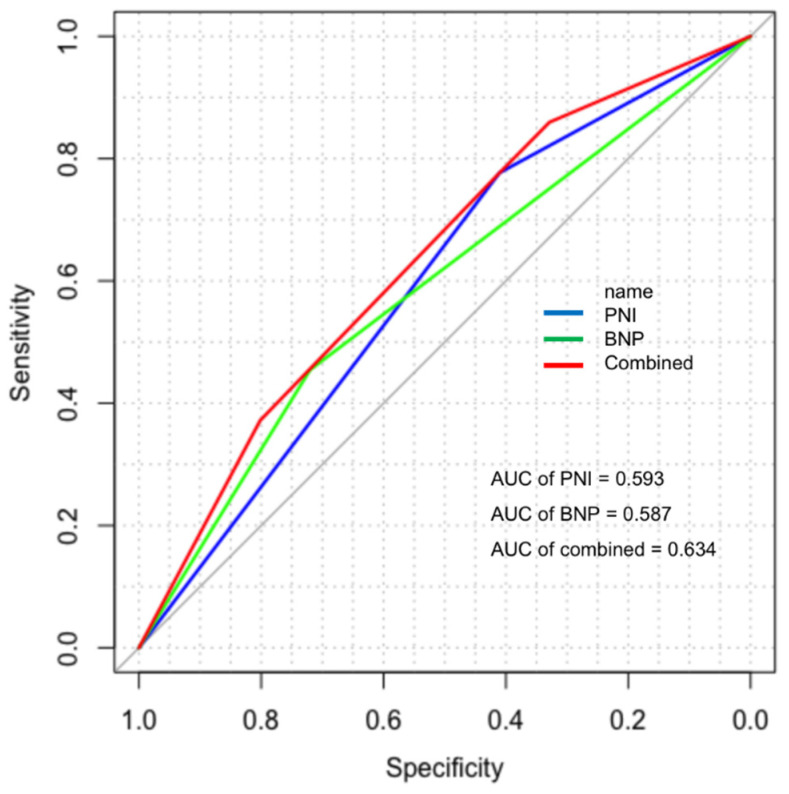
ROC curve for PNI, BNP, and combined indicator.

**Figure 5:**
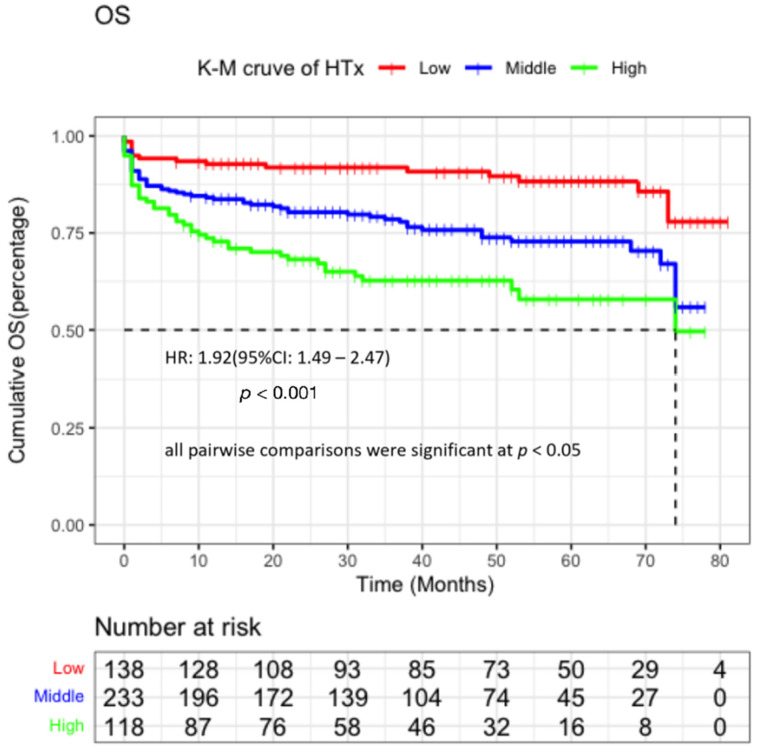
KM curve for combined indicators.

Next, we attempted to evaluate the effect of predictive potency blood biomarkers on survival. C-index test was used to analyze the data after matching the sensitivity, specificity, AUC, and concordance index of PNI, which were 77.7%, 41%, 0.594, and 0.593 (0.554–0.634), respectively. As for BNP, the results respectively were 72%, 45.5%, 0.587, 0.582 (0.536–0.628) (Table 4). The ROC curve for PNI, BNP, and the combined indicator showed that PNI had a superior AUC value than BNP (0.584 and 0.587). Meanwhile, the potency of the combined prediction of PNI and BNP was higher than that of single prediction (AUC = 0.634, c-index = 0.632, 95% CI: 0.585–0.680) (Figure 4). The effects of PNI and BNP at peri-transplant period, 1 year, and 5 year were shown in the Supplementary Materials. As shown in Figure 5, KM analysis with lowest combined index had the best overall survival than other subgroup, the 1-year survival rate of each subgroup of combined index was 92.0%, 84.1%, and 73.7% from low to high. All pairwise comparisons were significant at *p* < 0.05.

## 4. Discussion

In the present study, we investigated whether nutritional status and preoperative heart failure were associated with OS in patients undergoing HTx. Since the clinical prognosis after HTx is not always predictable, it is important for the clinician to investigate risk factors and develop a prediction model. In this study, we aimed to explore the prognostic significance of immune-inflammatory indexes in patients underwent HTx. This study revealed that PNI and BNP were independent prognostic blood biomarkers for OS after HTx, especially in short-term survival. More significantly, combining BNP and PNI as a predicting tool for prognosis of HTx would be optimal in clinical practice.

PNI was first applied as an objective nutritional screening tool by Buzby et al. in 1980 [5], and it has been used as a nutritional biomarker to predict the prognosis in some cardiovascular diseases such as acute heart failure, coronary artery disease, and myocardial infarction [2,3,4]. However, there is no research to assess the role of PNI in HTx. Malnutrition is a physical condition comprising the reduction in calories, protein, and micro-nutrients and, consequently, leads to the weakness of immune defenses [6]. Patients waiting for HTx are always in end-stage HF status, and gastrointestinal congestion caused by HF leads to a loss of appetite [7]. Thus, it is not a rare condition in the patients waiting for HTx. Shirley et al. have applied three scoring systems to assess the nutrition status among outpatients with heart failure, including geriatric nutritional risk index (GNRI), controlling nutritional status (CONUT) score, and prognostic nutritional index (PNI) [8,9,10]. Moreover, the results suggested malnutrition was a common condition among outpatients with HF and had a significant relationship with increased mortality [11]. In the present study, our results demonstrated lower PNI level was associated with worse survival, which was consistent with previous research. Recipients with lower PNI level tended to be older and had a relatively higher rate of using preoperative assist devices and cardiac drugs, which would increase mortality and morbidity after HTx.

BNP is associated with cardiac dysfunction and worse hemodynamic parameters, and it is a common blood biomarker for diagnosis and prognosis in many cardiovascular diseases such as heart failure and coronary heart disease [12]. Left ventricular ejection fraction is widely used to assess the cardiac function, but Gardner et al. demonstrated *n*-terminal-pro-BNP performed better in the prognostic prediction of HF than left ventricular ejection [13,14]. Moreover, there were some other studies focusing on the association between postoperative BNP level or donor BNP level and prognosis after HTx [15,16]. However, less attention was paid to investigate whether preoperative BNP level was able be the biomarker in the prognosis prediction. In the present study, univariable and multivariable analysis showed that BNP was an independent risk factor for overall survival (*p* = 0.024). Moreover, patients in higher BNP level groups had some characteristics such as older age, lower BMI, more preoperative IABP use, more preoperative ACEI use, and more preoperative dopamine use. Since age and waiting time on list have been proved to be independent risk factors in univariable and multivariable analysis, these factors would probably be responsible for worse survival in higher BNP level group. However, the present study implemented PSM to minimize confounding effects, and it would enhance the interpretability of the result that BNP is an independent indicator for OS after HTx.

Currently, there are some available score systems to assess the risk of cardiovascular surgery, such as EuroSCORE, EuroSCORE II, STS-SCORE, and so on [17,18,19]. Nevertheless, these scoring systems do not specially target the survival after heart transplantation. Thus, this study was conducted to investigate a simple risk prediction model that would be considered in clinical practice. In the present study, both PNI and BNP showed good diagnostic accuracy for the survival of HTx, especially in short-term survival, and the combined accuracy and validity of the two indicators were better than that of a single index. These findings extend and corroborate previous work in which evaluating PNI and BNP together was a potential clinical preoperative intervention target in forecasting the prognosis of patients underwent HTx.

## 5. Conclusions

Our study confirmed the clinical value of PNI and BNP as a screening tool in predicting the prognosis of heart transplantation since low PNI level and high BNP level are associated with poor survival. Moreover, the combined index showed better efficiency in predicting survival, especially short-term survival, and if it was poor, more targeted treatment should be applied to improve patients’ physical conditions before transplantation.

## Figures and Tables

**Table 1:** Baseline patient characteristics based on PNI and BNP.

Variables	Case (*n* = 489)	PNI			BNP		
		Low (*n* = 311)	High (*n* = 178)	*p*-Value	Low (*n* = 331)	High (*n* = 158)	*p*-Value
**Demographic Index**							
Sex				0.747			0.519
Male	383	245	138		262	121	
Female	106	66	40		69	37	
Age	47.57 ± 12.65	48.77 ± 11.97	45.47 ± 13.52	0.005	46.70 ± 12.68	49.38 ± 12.42	0.029
diagnosis				0.058			0.137
Ischemic cardiomyopathy	95	64	31		70	25	
Non-ischemic cardiomyopathy	308	190	118		197	111	
Congenital heart disease	19	8	11		15	4	
Other heart diseases	67	49	18		49	18	
recipient blood-type				0.707			0.023
A	164	109	55		111	53	
B	134	86	48		78	56	
AB	31	18	13		22	9	
O	160	98	62		120	40	
recipient BMI	23.00 ± 7.46	22.95 ± 8.74	23.07 ± 4.42	0.874	23.54 ± 8.63	21.87 ± 3.80	0.020
recipient/donor BMI	1.04 ± 0.26	1.05 ± 0.26	1.02 ± 0.28	0.131	1.02 ± 0.27	1.08 ± 0.25	0.016
recipient/donor age	0.81 ± 0.40	0.78 ± 0.35	0.87 ± 0.46	0.012	0.83 ± 0.41	0.78 ± 0.36	0.17
recipient/donor sex				0.951			0.703
Male/Female	35	22	13		25	10	
Male/Male	347	222	125		237	110	
Female/Male	78	50	28		52	26	
Female/Female	29	17	12		17	12	
recipient/donor blood-type				0.627			0.133
identical	400	252	148		277	123	
different	89	59	30		54	35	
Heart surgery history (Yes)	132	89	43	0.286	90	42	0.888
		190	99				
Charlson Comorbidity Index				0.582			0. 510
1	134	84	50		87	47	
2	60	40	20		44	16	
≥3	16	12	4		11	5	
waiting time		29.64 ± 15.56	29.31 ± 11.51	<0.001	29.98 ± 14.03	28.56 ± 14.57	0.955
**Preoperative Therapy**							
preoperative IABP	8	6	2	0.5	1	7	0.001
preoperative ECMO	6	4	2	0.875	2	4	0.070
preoperative ARB	85	54	31	0.988	58	27	0.906
preoperative ACEI	163	86	77	<0.001	123	40	0.009
preoperative dopamine	291	198	93	0.013	180	111	0.001
preoperative BB	381	233	148	0.035	264	117	0.155
**Preoperative Blood Index**							
Hb	133.15 ± 23.34	128.71 ± 23.57	140/90 ± 20.51	<0.001	134.37 ± 24.70	130.56 ± 19.67	0.093
ALT	71.19 ± 295.91	74.32 ± 249.15	65.72 ± 364.21	0.758	66.58 ± 289.29	80.85 ± 310.03	0.618
AST	60.25 ± 253.23	62.76 ± 248.85	55.88 ± 261.37	0.773	53.43 ± 211.17	74.54 ± 324.03	0.389
D-dimer	6.77 ± 7.81	6.18 ± 7.70	7.79 ± 7.93	0.029	6.42 ± 7.92	7.49 ± 7.55	0.161
troponin	1034.23 ± 5713.50	1029.75 ± 5154.90	1042.06 ± 6592.53	0.982	1135.31 ± 6313.58	822.47 ± 4197.02	0.572
Cr	99.24 ± 52.38	103.37 ± 60.19	92.04 ± 33.70	0.021	95.56 ± 47.65	106.96 ± 60.55	0.024
RBC	4.50 ± 1.53	4.37 ± 1.82	4.73 ± 0.78	0.013	4.51 ± 0.79	4.49 ± 2.45	0.916
PLT	180.45 ± 66.77	174.21 ± 70.21	191.35 ± 58.91	0.006	182.86 ± 65.63	175.39 ± 69.05	0.248
WBC	6.79 ± 4.65	6.38 ± 2.84	7.51 ± 6.69	0.010	6.93 ± 5.29	6.51 ± 2.89	0.355
triglyceride (TG)	1.12 ± 0.69	0.97 ± 0.47	1.39 ± 0.90	<0.001	1.19 ± 0.75	0.98 ± 0.52	0.002
LDL	2.08 ± 0.93	2.03 ± 0.92	2.16 ± 0.94	0.153	2.12 ± 0.94	2.00 ± 0.89	0.182

**Table 2:** Univariate and multivariate analysis of overall survival in patients with heart transplantation.

Variables	Univariate Analysis			Multivariate Analysis		
	HR	95%CI	*p* Value	HR	95%CI	*p* Value
**Demographic index**						
Sex	1.501	1.010–2.232	0.045	1.243	0.807–1.913	0.324
Age	1.027	1.011–1.043	0.001	1.025	1.009–1.042	0.002
Diagnosis	1.064	0.910–1.243	0.437			
Recipient blood-type	1.106	0.920–1.331	0.283			
Recipient BMI	1.014	0.989–1.040	0.287			
Recipient/donor BMI	1.372	0.695–2.707	0.362			
Recipient/donor age	0.662	0.401–1.093	0.107			
Recipient/donor sex	0.851	0.659–1.098	0.215			
Recipient/donor blood-type	1.226	1.013–1.485	0.037	1.118	0.936–1.337	0.220
Cardiac surgery history (Yes)	1.308	0.890–1.921	0.172			
Charlson Comorbidity Index	1.124	0.944–1.338	0.19			
Waiting time	1.013	1.001–1.024	0.036	1.014	1.003–1.026	0.012
**Preoperative therapy**						
Preoperative IABP	3.374	1.239–9.187	0.017	2.185	0.781–6.113	0.136
Preoperative ECMO	1.014	0.141–7.274	0.989			
Preoperative ARB	1.368	0.866–2.160	0.179			
Preoperative ACEI	0.506	0.331–0.773	0.002	0.675	0.431–1.059	0.087
Preoperative dopamine	1.553	1.063–2.270	0.023	1.339	0.901–1.988	0.148
Preoperative BB	0.704	0.475–1.044	0.081			
**Preoperative Blood index**						
Hb	0.987	0.979–0.995	0.002	0.997	0.987–1.007	0.521
ALT	1.000	0.999–1.001	0.884			
AST	1.000	1.000–1.001	0.433			
D-dimer	1.017	0.995–1.041	0.137			
Troponin	1.000	1.000–1.000	0.668			
Cr	1.002	1.000–1.005	0.082			
RBC	0.774	0.611–0.982	0.035	0.993	0.881–1.119	0.913
PLT	0.998	0.995–1.001	0.235			
WBC	1.014	0.990–1.039	0.242			
Triglyceride (TG)	0.734	0.544–0.990	0.043	0.932	0.670–1.296	0.675
LDL	1.111	0.910–1.357	0.3			
PNI	0.416	0.271–0.642	<0.001	0.613	0.378–0.993	0.047
BNP	1.917	1.340–2.743	<0.001	1.542	1.057–2.248	0.024

**Table 3:** Propensity score matching analysis of patients with heart transplantation based on PNI and BNP.

Variables	PNI (after PSM) (*n* = 232)			BNP (after PSM) (*n* = 252)		
	Low (*n* = 116)	High (*n* = 116)	*p* Value	Low (*n* = 126)	High (*n* = 126)	*p* Value
**Demographic index**						
Sex			0.872			0.479
Male	91	92		94	99	
Female	25	24		32	27	
Age	45.99 ± 12.29	46.18 ± 13.84	0.912	47.50 ± 12.35	48.45 ± 12.33	0.541
Diagnosis			0.913			
Ischemic cardiomyopathy	20	20		21	21	
Non-ischemic cardiomyopathy	75	79		85	85	
Congenital	5	4		4	4	
Other	16	13		16	16	
Recipient blood-type			0.892			
A	31	36		48	38	
B	36	32		28	45	
AB	9	9		10	8	
O	40	39		40	35	
Recipient BMI	23.04 ± 4.24	22.80 ± 4.57	0.667	22.41 ± 4.66	22.30 ± 3.60	0.843
Recipient/donor BMI	1.04 ± 0.26	1.04 ± 0.28	0.890	1.06 ± 027	1.04 ± 0.20	0.453
Recipient/donor age	0.89 ± 0.40	0.84 ± 0.38	0.363	0.79 ± 0.37	0.77 ± 0.34	0.646
Recipient/donor sex			0.449			0.821
Male/Female	4	9		9	10	
Male/Male	85	84		86	88	
Female/Male	23	18		25	20	
Female/Female	4	5		6	8	
Recipient/donor blood-type			0.714			
Identical	92	95		102	100	
Different	24	21		24	26	
Heart surgery history (Yes)	36	30	0.383	26	28	0.759
Charlson Comorbidity Index			0.902			
0	72	68		81	73	
1	27	32		27	37	
2	14	13		12	12	
≥3	3	3		6	4	
Waiting time	31.43 ± 15.72	29.27 ± 11.79	0.237	30.06 ± 14.05	28.75 ± 14.60	0.487
**Preoperative therapy**						
Preoperative IABP	1	1	1.000	0	1	0.316
Preoperative ECMO	2	1	0.561	1	0	0.316
Preoperative ARB	16	20	0.468	24	21	0.622
Preoperative ACEI	50	47	0.690	37	38	0.890
Preoperative dopamine	63	64	0.895	83	82	0.762
Preoperative BB	93	91	0.746	100	98	0.759

**Table 4:** Diagnostic evaluation of PNI and BNP in OS of Patients with heart transplantation.

Evaluation Index	PNI	BNP	Combined
Sensitivity (%)	77.7	72.0	85.1
Specificity (%)	41.0	45.5	34.2
AUC	0.594	0.587	0.634
C-index	0.593 (0.554–0.634)	0.582 (0.536–0.628)	0.632 (0.585–0.680)

## Data Availability

All data are included in this manuscript.

## Supplementary Materials

The following supporting information can be downloaded at: https://www.mdpi.com/article/10.3390/jcdd9020040/s1, Figure S1 PSM distribution for PNI and BNP. Table S1 Diagnostic Evaluation of PNI and BNP in peri-transplant period survival. Table S2 Diagnostic Evaluation of PNI and BNP in 1-year survival. Table S3 Diagnostic Evaluation of PNI and BNP in 5-year survival.
