# Supplementary material for: Combining Prognostic Nutritional Index and Brain Natriuretic Peptide as a Predicting Tool for Heart Transplantation
Source: J Cardiovasc Dev Dis. 2022 Jan 24;9(2):40. doi: 10.3390/jcdd9020040 (PMC8879512; doi:10.3390/jcdd9020040)
Supplement: Supplementary file 1 [file jcdd-09-00040-s001.zip › jcdd-1488065-supplementary.pdf]

Supplementary Materials:

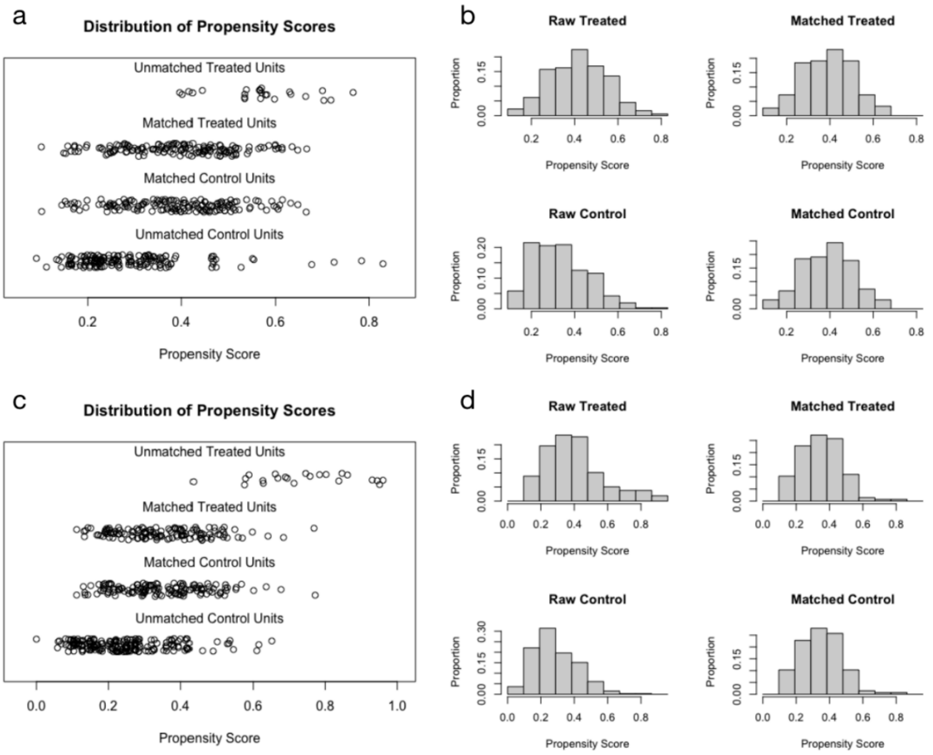

Figure S1 PSM distribution for PNI and BNP. The density of propensity scores was plotted as scatter diagram (a, c) and histogram (b, d) for each study arm before and after matching. Those matched showed a balanced and heterogeneous distribution in their likelihood of belonging to either study arm.

Table S1 Diagnostic Evaluation of PNI and BNP in peri-transplant period survival

Evaluation index	PNI	BNP	Combined
Sensitivity (%)	79.5	72	89.7
Specificity (%)	41.0	46.2	65.8
AUC	0.603	0.591	0.620
C-index	0.623(0.576-0.666)	0.597(0.538-0.656)	0.667(0.611-0.723)

Table S2 Diagnostic Evaluation of PNI and BNP in 1-year survival

Evaluation index	PNI	BNP	Combined
Sensitivity (%)	75.6	71.7	87.8
Specificity (%)	41.4	59.0	62.3
AUC	0.581	0.554	0.610
C-index	0.638(0.584-0.692)	0.563(0.479-0.647)	0.666(0.591-0.741)

Table S3 Diagnostic Evaluation of PNI and BNP in 5-year survival

Evaluation index	PNI	BNP	Combined
Sensitivity (%)	79.6	71.7	85.8
Specificity (%)	35.7	46.9	29.4
AUC	0.532	0.501	0.618
C-index	0.546(0.425-0.667)	0.492(0.384-0.601)	0.538(0.423-0.652)
